# Suprapatellar tibial fracture nailing is associated with lower rate for acute compartment syndrome and the need for fasciotomy compared with the infrapatellar approach

**DOI:** 10.1186/s10195-024-00749-3

**Published:** 2024-01-28

**Authors:** Essi E. Honkonen, Jussi P. Repo, Heidi Lehtokangas, Emma Luoma, Mikko Uimonen, Sami Nurmi, Antti Ylitalo, Antti Riuttanen, Tiia Kivelä, Ville M. Mattila, Piia Suomalainen

**Affiliations:** 1 Unit of Musculoskeletal Surgery, Department of Orthopaedics and Traumatology, Tampere University Hospital, Elämänaukio 2, PL 272, 33521 Tampere, Finland; 2 Department of Surgery, Central Finland Central Hospital, Nova Hospital, Hoitajantie 3, 40620 Jyväskylä, Finland

**Keywords:** Acute compartment syndrome, Fasciotomy, Suprapatellar approach, Infrapatellar approach, Tibial saft fracture, Intramedullary nail

## Abstract

**Background:**

Intramedullary tibial nailing (IMN) is the gold standard for stabilizing tibial shaft fractures. IMN can be performed through an infra- or suprapatellar approach.

**Purpose:**

The aim of this study is to compare the rate of fasciotomies for acute compartment syndrome between infra- and suprapatellar approaches.

**Methods:**

A total of 614 consecutive patients who were treated with IMN for tibial fracture between October 2007 and February 2020 were included in the study. The approach used for IMN was determined by the operating surgeon. Infrapatellar IMN was performed with the knee in deep flexion position, with or without calcaneal traction. Suprapatellar IMN was performed in straight or semiflexed position. The diagnosis of compartment syndrome was based on clinical analysis, but for some patients, a continuous compartment pressure measurement was used. The primary outcome was the rate of peri- and postoperative compartment syndrome treated with fasciotomies.

**Results:**

The study sample included 513 patients treated with infrapatellar IMN and 101 patients treated with suprapatellar IMN technique. The mean age of the patients was 44.7 years (infrapatellar technique) and 48.4 years (suprapatellar technique). High energy trauma was seen in 138 (27%) patients treated with infrapatellar technique and in 39 (39%) patients treated with suprapatellar technique. In the suprapatellar group (*n* = 101), there were no cases of peri- or postoperative compartment syndrome treated with fasciotomies. In the infrapatellar group (*n* = 513), the need for fasciotomies was stated in 67 patients, 31 patients (6.0%) perioperatively and in 36 patients (7.0%) postoperatively. The rate of fasciotomies (0/101 versus 67/513 cases) differed significantly (*p* < 0.001). There were no significant differences in the fracture morphology or patient demographics between the study groups.

**Conclusions:**

The suprapatellar technique is recommended over the infrapatellar approach in the treatment of tibial shaft fractures. The rate of peri- and postoperative compartment syndrome and the need for fasciotomies was significantly lower with the suprapatellar technique. The major cause of increased rate of peri- or postoperative acute compartment syndrome with infrapatellar IMN technique is presumably associated with the positioning of the patient during the operation.

**Level of evidence:**

3.

## Introduction

Tibial shaft fractures account for approximately 2% of all adult fractures [1, 2]. A potentially devastating complication, acute compartment syndrome (ACS) is reported in 1.2–11.4% of tibial shaft fractures [3–8]. ACS is a destructive end-point condition where the pressure in muscle compartments [increased intracompartmental pressure (ICP)] might cause muscular and nervous breakdown with very poor longterm outcome. The risk factors for ACS include male gender, open tibial fracture, high energy trauma, knee dislocation, age below 55 years, vascular injury in the same leg, Injury Severity Score (ISS) > 16, and polytrauma [3, 5, 7–11].

Limb swelling, caused by the injury itself, can lead to increased ICP in all four muscle compartments of the lower leg. Also, the operative treatment for the fracture can further intensify the ICP and, therefore, lead up to the development of severe ACS. The devastating cascade from increased ICP to the development of ACS is caused by decreased blood flow within the compartments, progressive ischemia and hypoxia, and eventually, if left untreated, muscle and nervous necrosis [5, 11].

The symptoms of ACS in the lower limb include severe pain, nerve palsy, paresthesia and paresis distal to the knee, and lack of arterial supply [5, 11]. The diagnosis of ACS is primarily based on the clinical estimation of the symptoms, but the measurement of ICP can be utilized as a support for the decision making [11, 12]. The gold standard for the treatment of ACS is immediate fasciotomies of all four muscle compartments, commonly performed during the initial stabilization of the fracture. Fasciotomies are usually carried out through the double incision technique, although a single incision technique can also be used [5, 13–15]. Skin closure after fasciotomies can be done directly or by using split thickness skin grafts [16, 17].

ACS and fasciotomies after tibial shaft fracture are associated with a higher risk for complications and poor functional outcomes [3, 6, 10, 18]. Fasciotomies can impair the fracture healing process, leading to longer healing times and increased rates of delayed union or nonunion (55% versus 17.8%) [18]. In cases of delayed ACS diagnosis, it has been reported that 10 out of 11 patients have ongoing problems, such as infections, sensory deficits, muscle weakness, and contractures [3, 10].

Currently, reamed intramedullary tibial nailing (IMN) is the standard method for stabilizing tibial shaft fractures [18]. Commonly used and widely reported operative techniques are performed through the infrapatellar (IP) or suprapatellar (SP) approaches [20–23]. Also, lateral or medial parapatellar approaches can be used. The incidence of tibial fracture nailing, according to the Finnish Care Register for Health Care, during the last 18 years has been approximately 10/100,000 persons per year [24].

Infrapatellar IMN is performed in supine position, with the knee in deep hyperflexion to accommodate the proper entry for the tibial nail. Reduction of the fracture is achieved with calcaneal traction, where a K-wire through the calcaneal bone is attached to the traction table. Instead, suprapatellar IMN is performed with the knee in full extension or only in 20–30° flexion. Fracture reduction is attained with straight pull of the limb by assisting operator. This traction method might be considered gentler when the intensity and duration of traction can be modified during the operation.

According to recent studies and a meta-analysis, SP IMN has multiple advantages compared with IP IMN [25]. These advantages include shorter fluoroscopy time, less anterior knee pain, better or similar recovery of knee function, and more accurate fracture reduction compared with the IP technique [16, 19, 25–30]. However, surgical time, blood loss, knee infection rate, nonunion rate, and closed reduction rate do not seem to differ significantly [19, 25–31].

There is some evidence that calcaneal traction for the IP IMN technique can lead to increased ICP in tibial fractures during intramedullary nailing [14, 32]. The injury itself and the use of traction might together increase the risk for ACS [14]. It has also been suggested that the deep flexion position of the leg in the IP technique might cause increased ICP by impairing venous drainage. Furthermore, the venous outflow from the injured limb might also be compromised by popliteal support, which is often mandatory to gain proper traction when using IP nailing technique. Traction, flexed position of the knee and popliteal support used in the IP IMN technique might be considered the main perioperative risk factors for the development of ACS.

A few studies have reported the rates of fasciotomies due to ACS after tibial fractures. Lindvall et al. reported of series of 22 patients treated with IP IMN with no fasciotomy [33]. Cheng et al. had 152 patients treated with SP IMN, of which one patient developed ACS and needed fasciotomies postoperatively [34]. In a multicenter case series of 180 patients treated with IP IMN, the risk of ACS was 3.8% [35]. To our knowledge, there is no previous studies that have compared the rate of fasciotomies between the SP and IP techniques.

The primary aim of this study is to compare the rate of peri- and postoperative fasciotomies for ACS using the SP IMN and IP IMN techniques in the treatment of tibial shaft fractures.

## Materials and methods

The study is a retrospective consecutive patient series conducted at a level I trauma center in Tampere University Hospital, Unit of Musculoskeletal Surgery, Finland. The study was approved by the institutional review board. In Tampere University Hospital, Unit of Musculoskeletal Surgery, Finland, ethical approval is not required for register-based studies (Medical Research Act, 488/1999).

**Fig. 1:**
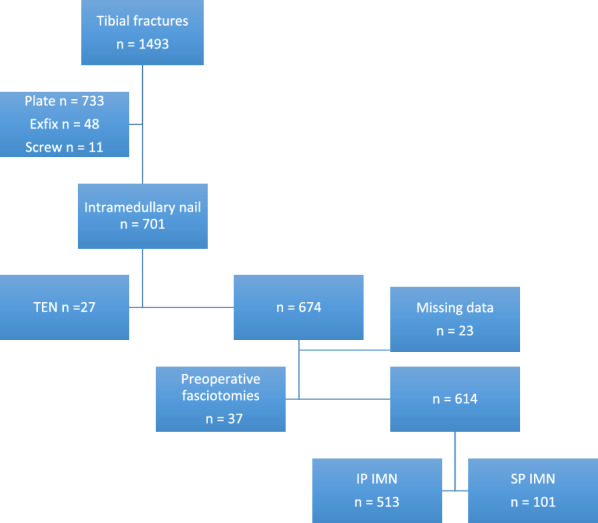
The flow chart. *IMN* intramedullary nail, *TEN* titanium elastic nail, *IP* infrapatellar nail, *SP* suprapatellar nail

Our institution is a tertiary referral trauma center with a catchment population of 0.9 million people. The treatment protocol of tibial shaft fractures, as well as most of the distal and proximal extra-articular metaphyseal tibial fractures, is intramedullary nailing. In the present study, we included all patients with a tibial fracture and closed epiphyseal plates who were treated with intramedullary nailing within 1 week of trauma between October 2007 and February 2020. Eligible patients were identified using a computer-based search with ICD-10 codes S82.1, S82.2, or S82.3 of the hospital’s electronic patient records. Patients with open growth plates or fixation with elastic intramedullary nail were excluded from the study. If the decision to perform fasciotomy was made before intramedullary nailing, the patient was also excluded (Fig. 1).

Patient records were reviewed and patient demographics, injury mechanism and energy, date of the injury, fracture pattern, date(s) of operation(s), time to definitive fracture fixation, the method of the operation (supra- or infrapatellar nailing), the need of fasciotomy and when the decision to perform them was made (pre-, peri-, or postoperatively), and anesthesia method were recorded. Traffic collisions and falls from heights greater than 2 meters were classified as high and other mechanisms as low energy injuries (Table 1).

**Table 1:** Sociodemographic and clinical details of the patients included in the study

	IP *n* = 513	SP *n* = 101	Significance
Mean (SD) age, years	44.7 (18.4)	48.4 (19.4)	*p* = 0.067
Age range, years	13–99	13–90	
Male, *n* (%)	344 (67)	60 (59)	*p* = 0.168
Fracture location			*p* = 0.532
Proximal tibia, *n* (%)	20 (4)	6 (6)	
Mid shaft, *n* (%)	242 (47)	50 (50)	
Distal tibia, *n* (%)	251 (49)	45 (45)	
Open fracture	*n* = 107 (21%)	*n* = 24 (24%)	*p* = 0.364
GI, *n* (%)	61 (57)	11 (46)	
GII, *n* (%)	18 (17)	7 (29)	
GIII, *n* (%)	28 (26)	6 (25)	
High-energy trauma, *n* (%)	138 (27)	39 (39)	*p* = 0.018

*IP* infrapatellar approach, *SP* suprapatellar approach, *G* Gustilo and Anderson classification [36, 37]

The primary care of the patients followed the algorithm principles of the Advanced Trauma Life Support (ATLS) guidelines [35]. Closed fractures without compartment syndrome were usually operated within 24 h after admission, whereas open fractures and fractures with compartment syndrome were operated as soon as possible (fasciotomies within 3 h and the initial fracture fixation and debridement of open fractures within 8 h). Open fractures were classified according to the Gustilo and Anderson system (GI, GII, GIIIA, GIIIB, and GIIIC) and fracture types according to OTA/AO classification [35–37]. Altogether, 78 different orthopedic surgeons and residents performed the operations during the study period. The level of expertise and the number of the operating surgeons per year remained at equivalent level. The residents always operated under the supervision of an experienced orthopedic surgeon.

The diagnosis of compartment syndrome was based on the clinical assessment of the orthopedic surgeon (i.e., tense muscle compartments, abnormal pain in the calf, painful passive calf muscle stretching, and/or sensory disturbances) [38–40]. Continuous compartment pressure measurement (CCPM) was addressed to those patients with an altered level of consciousness (i.e., head trauma or sedation) and in cases where compartment syndrome could not reliably be excluded by clinical examination.

As soon as the clinical suspicion of compartment syndrome was confirmed, fasciotomies were performed in a standardized manner using a two-incision technique to open all four compartments with long skin incisions. After the procedure, the fasciotomy wounds were left open with damp dressings. The closure of the fasciotomies was performed when clinical conditions were optimal. Split-thickness skin grafts were used when necessary.

Traditionally, in our hospital, IMN was performed using the infrapatellar approach with popliteal support and the knee in deep flexion position and with or without calcaneal traction. The suprapatellar IMN approach was first introduced at our hospital in 2017. SP IMN was performed with the knee in straight or semiflexed position. Well-known problems related to the IP approach and the amount of peri- or postoperative fasciotomies was raising concerns, and on the other hand, promising outcomes and the usefulness of SP approach were encouraging the experienced orthopedic specialists of our hospital to adopt SP IMN as a different surgical method. After introduction of the SP approach, the technique soon became the method of choice for most surgeons. Since October 2018, all but one nailing had been performed through the suprapatellar approach at our institution. This shift from IP technique to SP technique was the basis for formation of the study groups in this setting.

Data are presented as numbers with percentages (%) or means with standard deviation (SD). In statistical analysis, two-way tables were used with Pearson’s Chi-squared test with Yates’ continuity correction. Significance level was set to *p* < 0.05. The primary outcome was the rate of peri- and postoperative compartment syndrome treated with fasciotomies.

The reporting of the results adheres to the Strengthening the Reporting of Observational Studies in Epidemiology (STROBE) checklist [41].

## Results

A total of 1493 tibial fractures were treated at our institution between October 2007 and February 2020 (Fig. 1). The treatment for 674 cases was IMN. Fixation method in 27 cases was titanium elastic nail (TEN), and they were excluded. The decision to perform fasciotomies was made preoperatively (at any point before intramedullary nailing) in 37 cases, which were excluded because the need for fasciotomies was not affected by the chosen IMN technique. Of these remaining 614 patients, 513 were treated with IP IMN and 101 with SP IMN. Fractures and patient demographics did not differ significantly between the IP and SP groups (Table 1). Only difference between the groups was the amount of high energy trauma, in favor for suprapatellar nailing group (27 versus 39%, *p* = 0.018).

In the SP group, there were no fasciotomies performed peri- or postoperatively (0/101, 0%). In the IP group (*n* = 513), the need for fasciotomies was noted perioperatively in 31 out of 513 patients (6.0%) and postoperatively in 36 out of 513 patients (7.0%). The total rate of fasciotomies in IP group was 13.1% (67/513 patients). There was statistically significant difference between IP and SP groups (*p* < 0.001).

All fractures included in the analyses were classified using AO/OTA fracture classification method (Table 2) [35–37]. No significant differences could be detected between the study groups. The mechanism of the injury was determined as presented in Table 3. The study groups were comparable and statistically significant differences between the groups were not found.

**Table 2:** Fracture types according to AO/OTA classification [34–36]

Fracture classification, *n* (%)	IP *n* = 513	SP *n* = 101	*p* = 0.290
42–A1	219 (43)	31 (31)	
42–A2	69 (13)	15 (15)	
42–A3	51 (10)	8 (8)	
42–B1	63 (12)	19 (19)	
42–B2	52 (10)	15 (15)	
42–B3	15 (3)	4 (4)	
42–C1	18 (4)	3 (3)	
42–C2	11 (2)	4 (4)	
42–C3	15 (3)	2 (2)	

*IP* infrapatellar approach, *SP* suprapatellar approach. No statistical differences could not be detected between the study groups

**Table 3:** The mechanism of the injury

Mechanism of injury, *n* (%)	IP *n* = 513	SP *n* = 101	*p* = 0.275
*Traffic*			
Car	10 (2)	4 (4)	
Motorbike	36 (7)	4 (4)	
Bicycle	25 (5)	4 (4)	
Pedestrian	20 (4)	3 (3)	
*Fall*			
Same level	249 (49)	51 (51)	
Stairs/ < 1 m	35 (7)	5 (5)	
From heights > 1 m	16 (3)	4 (4)	
*Sports*			
Soccer, ice hockey, tennis, padel, etc.	32 (6)	3 (3)	
Skiing (cross country and slalom) and snowboarding	10 (2)	2 (2)	
Motor sports	8 (2)	2 (2)	
Martial arts	0 (0)	1 (1)	
Running, track and field, and athletics	1 (0)	0 (0)	
Cycling	2 (0)	0 (0)	
Other sport	15 (3)	6 (6)	
Other	54 (11)	9 (9)	

*IP* infrapatellar approach, *SP* suprapatellar approach. No statistical differences could not be detected between the study groups

## Discussion

The findings of this study show that there was a significant difference between the SP and IP IMN techniques in the rate of fasciotomies performed peri- or postoperatively after tibial fractures. Since the implementation of SP IMN into the treatment protocol of tibial fractures at our hospital, peri- or postoperative fasciotomies have not been required. To our knowledge, some benefits of using SP approach have been reported, but no previous studies are concerning ACS and the need for fasciotomies in relation to the nailing technique chosen [19, 25–30].

A major contributing factor causing compartment syndrome among patients treated with IMN for tibial fracture is presumably associated with the positioning of the patient during the operation. In the SP technique, the patient is supine with a raised lower extremity, and the knee is either straight or semiflexed. The venous outflow is not compromised during the operation. In contrast, in the IP technique, the knee is deep flexion, which can remarkably hinder venous outflow. Further, popliteal support is used with IP technique. This can further progress to ACS through increased ICP and decreased blood flow within the compartment [5, 11]. There is also some evidence that calcaneal traction leads to increased ICP in association of tibial fractures during intramedullary nailing [14, 31]. Calcaneal traction is commonly used in IP IMN but not in SP IMN, which may be one factor contributing to the difference in the rate of fasciotomies. Some reports about alternative tibial IMN insertion techniques exist, including lateral and medial parapatellar intramedullary nailing. They both can be performed without traction and popliteal support and therefore might also be useful in avoiding ACS [42]. However, in our hospital, and therefore in this study, only IP and SP IMN techniques have been used. Nonetheless, the theory of compromised venous outflow, due to traction and popliteal support as a significant cause of peri- or postoperative fasciotomies, is reinforced by the results of our study.

Previously, fasciotomies might not have been considered as a complication of a surgical method but rather a treatment of compartment syndrome resulting from the fracture itself. To date, only a few studies have reported the rates of fasciotomies in association to the treatment of tibial fractures using an intramedullary nail [25, 32–34]. ACS and fasciotomies after tibial fracture are associated with a higher risk for complications, slower fracture healing, and poor functional outcomes [3, 7, 10, 18, 25]. Therefore, the suprapatellar approach can be recommended for reducing the rates of peri- and postoperative compartment syndrome requiring fasciotomies. Moreover, the use of the suprapatellar approach should lead to both a decrease in the morbidity associated with fasciotomies and better functional outcomes [19, 25–31].

The present study has several strengths. The sample size can be considered large enough to draw conclusions. Undoubtedly, an equivalent sample size in both groups with a larger sample of SP technique could have provided a more solid basis for statistical outcomes. However, as the suprapatellar technique has been used since 2017 at our hospital mostly as the only surgical method for tibial shaft fractures, we have large continuous and consecutive data of the SP technique as well.

The total rate of fasciotomies in our study was 16.2%, including preoperative fasciotomies. Acute compartment syndrome has been previously reported to associate with 1.2–11.4% of shaft fractures of the tibia [3, 6–8]. In our hospital, the decision of performing fasciotomies is based on clinical suspicion and the measurements of CCPM when needed. Due to the presumably devastating consequences of ACS, when suspected, fasciotomies have been performed without hesitation. This explains the slightly increased fasciotomy rate when compared with the previous literature. However, with similar diagnostic methods for ACS, after implementation of suprapatellar IMN technique, ACS was not suspected, and subsequently, no fasciotomies were performed. Further, we did not observe any increase in the number of missed compartment syndromes and postcompartment syndrome conditions treated at our institute.

One limitation of the study could be considered to be the large number of operating surgeons. The fact is that in a large and sparsely populated country like Finland, the tibial shaft fracture occurrence per hospital is relatively low. Additionally, every orthopedic surgeon on call in our hospital are experienced and are therefore able to perform intramedullary nailing in cases of tibial fracture. As a result, the annual number of tibial intramedullary nailing performed by one orthopedic surgeon is also low. However, the results of our study, in which the need of fasciotomies completely disappeared, support SP nailing as a safe alternative, and the outcome is not dependent on the operator.

In the present setting, the study groups differ in size due to the fact that SP nailing has become a standard procedure over IP nailing since 2017 in our hospital. Therefore, equal amount of SP nailing cases are not yet applicable. Nevertheless, the study groups are otherwise comparable, when considering patient age, gender, fracture type and pattern, and the mechanism of the injury. Only difference between the groups was the amount of high energy trauma, in favor of suprapatellar nailing group (27 versus 39%). Interestingly, even though the patients in SP group had a higher incidence of high energy trauma, they still had no peri- or postoperative fasciotomies performed. Again, we did not see any increase in the number of missed compartment syndromes and post compartment syndrome conditions treated at our institute.

Another limitation of the study is its retrospective nature. In theory, the results of the present retrospective study could be confirmed in a large-scale randomized controlled trial comparing these two approaches (IP and SP) with a primary endpoint of peri- and postoperative compartment syndrome. However, this kind of study design would be highly questionable, as the results of the current study clearly state the benefits of the SP technique compared with the IP technique in terms of the rate of fasciotomies performed. Instead, a large-scale register study with an even larger number of patients could confirm the results of the present study.

## Conclusions

Based on the findings of the present study, the suprapatellar intramedullary nailing technique used in the treatment of tibial shaft fractures notably decreases the rate of fasciotomies for peri- and postoperative compartment syndrome compared with the infrapatellar intramedullary nailing technique.

## Data Availability

The datasets used and/or analyzed during the current study are available from the corresponding author on reasonable request.

## Abbreviations

IMN: Intramedullary nailing
ACS: Acute compartment syndrome
ICP: Intracompartmental pressure
IP: Infrapatellar
SP: Suprapatellar
CCPM: Continuous compartment pressure measurement
